# G protein–coupled receptors: from radioligand binding to cellular signaling

**DOI:** 10.1172/JCI178109

**Published:** 2024-03-01

**Authors:** Howard A. Rockman, Robert J. Lefkowitz

**Affiliations:** 1 Department of Medicine,; 2 Department of Cell Biology, and; 3 Howard Hughes Medical Institute, Duke University School of Medicine, Durham, North Carolina, USA.

## Abstract

Radioligand binding techniques facilitated the identification and study of G-protein coupled receptors that now represent the largest class of targets for therapeutic drugs.

G protein–coupled receptors (GPCRs) represent by far the largest, most versatile, and ubiquitous class of cellular receptors, comprising more than 800 distinct receptors. They represent the largest class of targets for therapeutic drugs, comprising almost one-third of all FDA-approved agents, amounting to some 700 different drugs. Yet when one of us (Lefkowitz) began his career, there was no concrete evidence that drug and hormone receptors actually existed as independent molecular entities. And moreover, the tools did not exist to prove their existence and study their properties. All this changed in the early 1970s with the development of radioligand-binding techniques (1), which permitted the identification and study of receptors such as the β-adrenergic receptor (βAR) (2). Work on the β-2 adrenergic receptor (β2AR) would become the prototype for studies of this large receptor family.

## Strategies that advanced the study of GPCRs

Initial development of ligands for the βAR was done in model systems, such as frog and turkey erythrocyte membranes, which are rich sources of βARs, which are coupled to the enzyme adenylate cyclase to catalyze formation of the second messenger cyclic AMP. But very quickly thereafter, experiments were devised to determine whether these powerful methods could be extended to human material, which would enable clinically relevant investigations of receptor signaling and regulatory mechanisms. Two of the earliest such studies were published in the *JCI*, one dealing with the binding of [3H]-dihydroalprenolol to β2ARs located on human lymphocytes (3) and the other the binding of [3H]-dihydroergocryptine to α-adrenergic receptors located on human platelets (4). These studies, published almost 50 years ago, highlight the rigorous approaches that were necessary to validate that the binding of specific radioligands to membranes from cells was indeed representative of a true interaction with the relevant receptors and not binding to nonspecific sites.

In both studies, with lymphocytes and platelets, the cell type was selected because of its easy accessibility simply by venipuncture. Several criteria were established and met to validate that radioligand binding was in fact occurring at the physiologically relevant receptors. First, the binding was rapid, reversible, and of high affinity. Moreover, the affinity of radioligand binding closely matched the affinity of nonradioactive forms of the radioligand antagonists determined in pharmacological experiments to block agonist actions on the cells. Second, radioligand binding was saturable and to a discrete and low number of sites per cell, approximately 2,000 in the case of the lymphocyte β2AR and 200 for the α-adrenergic receptors on platelets. Finally, and perhaps most importantly, antagonist and agonist drugs competitively inhibited the binding of the radioligands with specificity and stereospecificity identical to those determined for these agents in exerting their pharmacological effects on the cells (3, 4).

These approaches made the direct study of GPCRs accessible in human material for the first time and were rapidly leveraged by many groups. For example, the lymphocyte βAR assay was used to study regulation of the receptors by desensitization after catecholamine use, as observed in asthma or in pheochromocytoma, and in diseases such as heart failure, hyperthyroidism, leukemia, dementia, and others (5). The α receptor assay was used to study their regulation in, for example, essential thrombocythemia and depression. It was also used to define the α2 receptor subtype found in platelets. And human platelets were eventually used as the source to purify the α2 receptor, which led to its cloning and the determination of its primary amino acid sequence, one of the very first GPCRs whose gene sequence was determined.

Radioligand binding became rapidly appreciated as one of the most basic core technologies for studying GPCRs of all types. Not only did this technique enable the purification and characterization of the receptors, but it led to numerous important discoveries in cell biology, physiology, and pharmacology. Prominent among these was the demonstration that, rather than representing static entities on the cell membrane, the number, properties, and even subcellular distribution of the receptors was strongly influenced by many factors, including hormones, other drugs, and various disease states.

Over the ensuing two decades, major advances were made in our understanding of the structure, activation, and inactivation of GPCR signaling through the cloning of the β2AR, revealing its seven-transmembrane structure (6), the discovery of a family of enzymes that phosphorylate agonist-stimulated βARs (7), and the subsequent discovery of the receptor adaptor protein β-arrestin (8), which, when recruited to agonist-occupied βARs, sterically interdicts G protein coupling, leading to the ubiquitous cellular phenomenon of receptor desensitization (9). Thus, by the early 1990s, the processes by which a GPCR is activated and then rapidly turned off were thought to be largely understood. As so often is seen in biology, Mother Nature had many surprises yet in store for us.

## A paradigm shift in the understanding of GPCR signaling

The discovery by the Ulrich laboratory that the Gq-coupled GPCRs for endothelin-1, lysophosphatidic acid, and thrombin (10, 11) could transactivate the receptor tyrosine kinase (RTK) epidermal growth factor receptor (EGFR) to initiate mitogen-activated protein (MAP) kinase signaling introduced a surprising new paradigm. The mechanism proposed at the time was that agonist stimulation of a Gq-coupled GPCR initiates an undefined intracellular signal to induce the extracellular activity of a membrane metalloproteinase (MMP) and triggers cleavage of the precursor ligand heparin-binding EGF (HB-EGF). Local release of HB-EGF then directly interacts with the ectodomain of EGFRs to stimulate a cascade of intracellular signaling. While this newly appreciated GPCR to RTK signaling was intriguing, the precise molecular details of how agonist-occupied GPCRs lead to HB-EGF release and subsequent EGFR signaling was not delineated.

Contemporaneously, the Lefkowitz laboratory identified a signaling paradigm by which the then recently discovered GPCR desensitizing proteins, β-arrestins 1 and 2, also function as adapter proteins that link GPCRs to MAP kinase growth regulatory pathways (12). Stimulation of β2ARs was shown to recruit β-arrestin to assemble a protein complex that includes the receptor β-arrestin and the nonreceptor tyrosine kinase c-Src, resulting in the activation of ERK MAP kinase signaling. The conceptual novelty of this discovery led to a paradigm shift in our understanding of GPCR signaling by showing that β-arrestin recruitment to agonist-occupied βARs not only functions to terminate receptor–G protein coupling, but in a second wave, activates β-arrestin–dependent MAP kinase signal transduction.

Merging these two concepts, Noma et al. in the *JCI* (13) set out to determine whether catecholamine stimulation of the dominant adrenergic receptor in the heart, the Gs-coupled β1-adrenergic receptor (β1AR), could transactivate EGFRs and if so, identify the molecular mechanism by which this pathway initiates signaling. In a series of in vitro and in vivo studies, Noma et al. showed that catecholamine-stimulated β1ARs recruit β-arrestin to GPCR kinase 5/6-phosphorylated receptors, an interaction that activates Src-MMP–triggered shedding of HB-EGF. Release of HB-EGF ligand stimulates EGFR phosphorylation and ERK activation. Moreover, activation of this β1AR/EGFR transactivation pathway provides cardioprotection in vivo under conditions of catecholamine toxicity, such as is observed in chronic heart failure. It was proposed at the time that it might be possible to identify or develop ligands that act as classical beta blockers for G protein signaling, while at the same time activate β-arrestin–mediated pathways to protect the heart in response to catecholamine stress (13).

**Figure 1:**
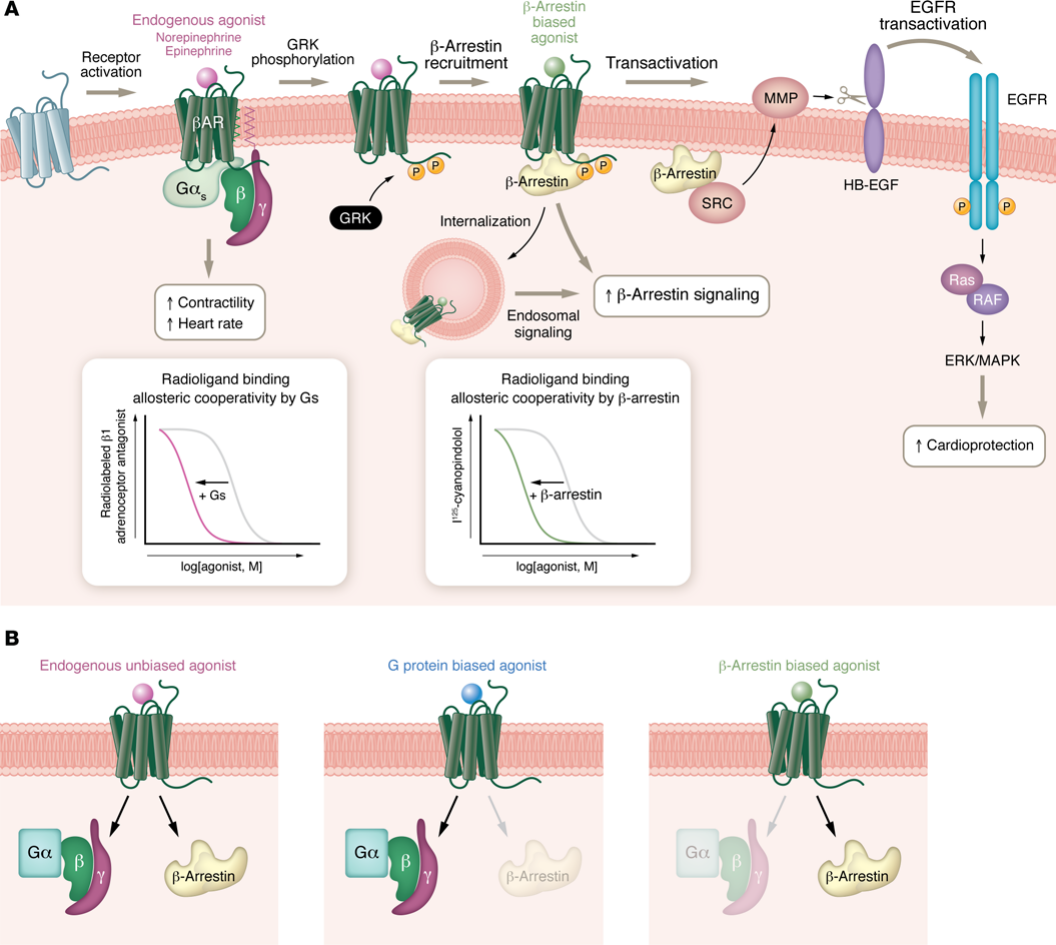
Current concepts in GPCR signaling. (**A**) The binding of norepinephrine to the orthosteric site of the βAR leads to the formation of a high-affinity ternary complex composed of agonist, βAR, and heterotrimeric G protein (including Gα, Gβ, and Gγ). Competitive radioligand-binding assays show shifted curves in the presence of G protein (Gs). A leftward curve shift indicates allosteric cooperativity and stabilization of a high-affinity receptor conformation. The high-affinity ternary complex stimulates G protein–mediated cAMP accumulation and intracellular signaling. As a physiological consequence, heart rate and contractility increase. β-Arrestins are recruited to agonist-occupied GPCR kinase (GRK) phosphorylated receptors to turn off, or desensitize, the G protein signal by sterically preventing G protein binding. β-Arrestin also stabilizes a high-affinity conformation of the βAR, as reflected by the leftward shift in the competition radioligand binding curve. β-Arrestin mediates receptor endocytosis and functions as a scaffold for many signaling proteins, thereby activating a suite of distinct β-arrestin–dependent signaling pathways. β-Arrestin–mediated signaling can occur inside the cell, initiated by the internalized receptor–β-arrestin complex, or at the plasma membrane via EGFR transactivation and ERK activation. Notably, the transactivation pathway is cardioprotective. (**B**) Biased signaling is a process whereby alternate GPCR ligands preferentially stimulate cellular pathways through differential engagement of a transducer, either G proteins or β arrestins, leading to distinct signaling profiles.

These findings encompassed several conceptual advances: first, that β-arrestin is not simply a desensitizing protein, but rather could also be a transducer of GPCR signaling; second, that signaling downstream from agonist-stimulated βARs could follow distinct pathways — either via Gs proteins (i.e. cAMP/PKA) or β-arrestin (Src-ERK and EGFR transactivation); third, that theoretically it should be possible to identify new ligands that would stabilize distinct receptor conformations that would exhibit preferential coupling to, and signaling through, either G proteins or β-arrestins, thus initiating distinct signaling profiles (known as “biased signaling”); and finally, that in the heart, activating GPCR–β-arrestin–mediated signaling would be cardioprotective under conditions of cardiac injury (Figure 1). Indeed, in the fullness of time, these mechanistic insights have been confirmed (14–18) and have been greatly expanded by recent structural and spectroscopic methods demonstrating the highly dynamic conformational nature of GPCRs when interacting with transducers and biased ligands (19).

On a more personal note, work on the two papers (3, 4) was led by students Lewis T. (Rusty) Williams, Kurt Newman, and Nanette Bishopric, all of whom, exposed to the excitement of biomedical research, would go on to distinguished academic careers. Williams was an MD/PhD student, and Newman and Bishopric were third-year medical students at the time they did this work.

## Version 1 03/01/2024

Electronic publication
